# Does m-health-based exercise (guidance plus education) improve efficacy in patients with chronic low-back pain? A preliminary report on the intervention’s significance

**DOI:** 10.1186/s13063-022-06116-z

**Published:** 2022-03-03

**Authors:** Fuming Zheng, Shufeng Liu, Shanshan Zhang, Qiuhua Yu, Wai Leung Ambrose Lo, Tingni Li, Chu Huai Wang

**Affiliations:** Department of Rehabilitation Medicine, The First Affiliated Hospital, Sun Yat-sen University, Guangzhou, 510080 China

**Keywords:** Low back pain, Health education, Mobile health, Exercise therapy

## Abstract

**Background:**

The utilization of mobile health (m-health) has rapidly expanded during the COVID-19 pandemic, and there is still a lack of relevant clinical data pertaining to chronic low-back pain (CLBP) management. This study was designed to compare the effectiveness of m-health-based exercise (via guidance plus education) versus exercise (via guidance) during CLBP management.

**Methods:**

Participants (*n* = 40) were randomly assigned to intervention and control groups. The intervention group received m-health-based exercise (via guidance plus education), whereas the control group received m-health-based exercise (via guidance). The exercise prescription video and educational content were sent to participants by the application (app), *Ding Talk*. Repeated-measures analysis of variance was used to test the baseline’s intervention effects, 6-week follow-up, and 18-week follow-up. We selected function (Roland and Morris Disability Questionnaire) and pain intensity (current, mean, and most severe Numeric Rating Scale in the last 2 weeks) as the primary outcomes, changes of negative emotion (depression, anxious), and quality of life as the secondary outcomes.

**Results:**

Time’s significant effect was found in pain, function, and health-related quality of life in both groups, but time did not show significant interaction effects. Participants were able to use m-based education with their anxiety and depression after treatment, but the relief only lasted until week 6. No differences were found on the aspect of mental health-related quality of life.

**Conclusion:**

Preliminary findings suggest that m-health-based exercise (via guidance) may be a convenient and effective method to treat CLBP. However, additional health education didn't help more. More rigorous controlled trials are needed to improve the therapeutic effect in future studies.

**Trial registration:**

Chinese Clinical Trials Registry Number ChiCTR2000041459. Registered on December 26, 2020.

## Background

Chronic low-back pain (CLBP) is a very common symptom that can occur in different age groups (from the young to the elderly) [1]. CLBP prevalence can start early in life [2]. According to the 2019 Global Burden of Disease Study, low-back pain is one of the top ten causes of the disability-adjusted life-year (DALY) in both the 10 to 24-year and 25 to 49-year age groups [3]. CLBP causes not only physical suffering but also psychological and social problems [4]. There are a wide range of interventions available for CLBP and international guidelines that emphasize the self-management approach based on a biopsychosocial model [5–7].

Core stability exercise (CSE) has been proven useful for CLBP within short-term durations [8]. However, patients with CLBP have poor adherence to exercise training without supervision, leading to poor long-term outcomes [9]. We suggest the reason is the lack of remote guidance and patient education after the patient leaves the hospital. Education for patients with CLBP plays a critical role in influencing patients’ self-management. Research shows that close communication and effective education can improve patients' ability to self-manage pain [10]. For example, the back school is a very effective method for alleviating CLBP [11]. However, most traditional education models are still based on a biomechanical approach; research has shown that the biopsychosocial model education seems to be more effective [12]. Additionally, medical resources are unevenly distributed in China [13]; as a result, patients in remote areas have difficulty in receiving accurate and scientific-educational information. With the emergence of mobile health (m-health), solutions to difficult problems are provided [14, 15]. A preliminary study suggested that application (app)-based patient self-management has a better effect [16]. The results of a systematic review indicated that teletherapy strategies can be effective in improving quality of life among persons with chronic pain; however, those trials tended to report lower adherence due to inefficient communication [17]. Since the beginning of 2020, COVID-19 began to globally spread, and its impact may last until 2025 [18]. The most effective method to control the epidemic is social isolation [19]. COVID-19 has influenced the rapid development of m-health [20]. The idea of developing a home-based fundamental approach to alleviating low-back pain during pandemic has been proposed [21], but it is unknown whether m-health-based exercise (via guidance plus education) improves efficacy in patients with CLBP.

Our study aim was to compare the effectiveness of m-health-based exercise (via guidance plus education) versus exercise (via guidance) during CLBP management. We hypothesized that m-health-based exercise (via guidance plus education) would improve function, life quality, pain relief, depression, and anxiety compared to m-health-based exercise (via guidance only), and education can also improve treatment adherence.

## Methods

### Overall design

This was a single-blinded, pilot parallel-group randomized (1:1) controlled trial. The participants were managed by the Ding Talk V.5.0, which is a smart mobile office platform specially created for global enterprises and organizations produced by Alibaba. It can be used for more effective communication and online data collection. The trial was prospectively registered with the Chinese Clinical Trials Registry Number: ChiCTR2000041459 (12/26/2020).

### Participants

The participants were recruited from October 4, 2020, to November 1, 2020, via WeChat, a social networking tool in China. Participants were screened via electronic questionnaire (provided by Ding Talk). Eligible subjects were invited to complete the pretrial questionnaire.

Participants were eligible for inclusion if they were between the ages of 18 and 65 and had CLBP within a minimum of a 12-week duration. Participants with back pain associated with a specific diagnosis (e.g., spinal stenosis, lumbar disc herniation, and lumbar fracture), who had difficulty in participation (e.g., unable to master the Ding Talk apps or unable to speak Chinese) or not interested in the trial, were excluded. A total of 52 people signed up, and we selected 40 eligible participants through detailed online consultation. All participants provided online informed consent for trial participation and electronic signatures for participation in treatment.

### Randomization and blinding

Following the baseline assessment, the 40 eligible participants were numbered and randomly divided into two groups by IBM SPSS statistics 22 to receive m-health-based exercise (via guidance; control group) or m-health-based exercise (via guidance plus education; intervention group). Participants were informed that the trial was comparing two different forms of online self-management exercise before allocation; they did not know each other’s treatment method. Since the outcomes were self-reported, bias from the assessors was avoided. The researchers were blind to the study.

### Interventions

The intervention time was 6 weeks; the participants were asked to complete the exercise at least 3-times per week. The exercise instruction was provided by a physiotherapist with 3 years of experience, and patient education was provided by a rehabilitation physician. All treatments were performed online.

#### M-health-based exercise guidance

**Fig. 1:**
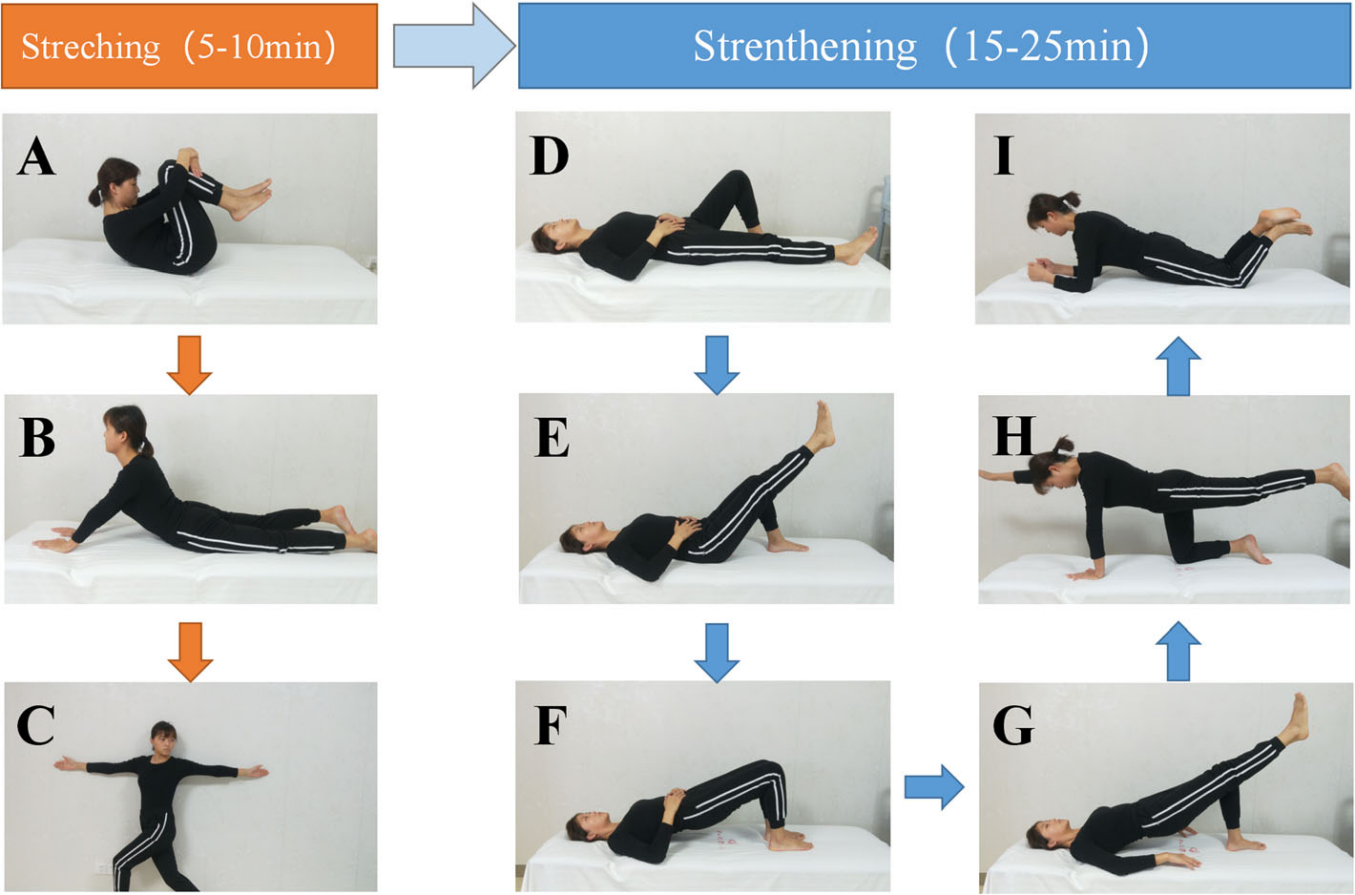
Exercise program. **A ** Rolling spine exercise, 1X60s. **B L**umbar extension 2X15s. **C** Lumbar rotation 4X15s. **D** Hallowing training 4X15s. **E** Hip-single leg support 4X15s. **F** Hip bridge 4X15s. **G** Hip-single leg support 4X15s. **H** Superman action 4X30s. **I** Half plank 4X30s

Both groups received exercise videos designed by physiotherapist, which included two sections: stretching and strengthening (Fig. 1). Stretching can relax tired superficial muscles, and strengthening can improve control and coordination of the spine and pelvis [22].

The first week’s main topic was to teach the participants how to use Ding Talk. We uploaded a daily-CSE video, and then we taught the participants how to record perception; for the weekends, we asked them to complete an exercise log to answer the following questions: (1) How’s the exercise going? (2) Do you have any questions about the interventions? (3) How is your CLBP changing?

For the next 5 weeks, the participants were asked to perform the exercise at least 3 times a week with a corresponding record. Each exercise lasted between 20 and 45 min, and we reminded the participants to finish the recording log over the weekend. After reviewing the recording logs, we replied to each comment, either by encouraging or answering question to maintain effective researcher-participant communication.

#### M-health-based patient education

Additionally, the intervention group received the CLBP-education online lessons. Previous studies have shown that pain science education can improve exercise adherence [23, 24].

In the first course week, the rehabilitation physician organized an online meeting for members to introduce themselves and share their experiences pertaining to low-back pain. After understanding the participants’ basic information, the weekly topics were formulated as follows: (1) Week 2: What is CLBP? (2) Week 3: How does CLBP appear? (3) Week 4: How does CSE work? (4) Week 5: Identify bad lifestyle habits, such as being sedentary. (5) Week 6: Conclusion and expectation. To ensure that every participant could receive educational information, we required weekly study report submissions on what participants learned for that week. The study report was open, and the group members could see each other. We encouraged group communication to form a positive social environment.

### Follow-up

The participants scanned the provided QR codes to complete the questionnaire at baseline (before randomization) and after randomization at week 6 (post treatment) and week 18. Participants were compensated with cash through combine lucky red envelopes (a random red envelope reward includes electronic cash of 1 to 5 yuan) for each follow-up.

### Outcome measurements

Sociodemographic and back pain information was obtained at baseline (Table 1). All primary and secondary outcomes were administered at each time point (baseline, week 6, week 18).

**Table 1:** Baseline characteristics of participants by treatment group

	Intervention group (*N* = 20)	Control group (*N* = 20)
Age, mean (SD)	34.0 (14.4)	34.9 (14.5)
Female, *n* (%)	14 (70)	12 (60)
BMI, mean (SD)	21.5 (2.7)	22.3 (3.6)
Working status, *n* (%)		
Employed	15 (75)	16 (80)
Unemployed/retired	5 (25)	4 (20)
Education level, *n* (%)		
Primary school or less	1 (5)	0 (0)
Junior	1 (5)	1 (5)
Senior	3 (15)	1 (5)
College or higher	15 (75)	18 (90)
Pain duration, *n* (%)		
≤ 1 year	6 (30)	5 (25)
< 5 years	7 (35)	11 (55)
≥ 5 years	7 (35)	4 (20)
RMDQ	4.8 (2.7)	4.0 (2.7)
AVERAGE NRS in the last 2 weeks	4.7 (1.8)	4.0 (1.7)
Current NRS	3.4 (1.8)	3.2 (1.8)
Most severe NRS in the last 2 weeks	6.6 (2.0)	6.2 (1.8)
SDS	47.9 (11.5)	41.6 (7.7)
GAD-7	4.7 (2.8)	3.3 (3.1)
SF-36 PCS	39.5 (6.4)	44.3 (7.7)
SF-36 MCS	48.3 (10.2)	51.7 (10.0)

#### Primary outcome

We selected two primary outcomes, the Roland Morris Disability Questionnaire (RMDQ) [25], which measures back pain disability (scale 0–24; higher scores indicate greater functional limitation), and the Numeric Rating Scale (NRS) [26], which measures mean pain intensity during the last 2 weeks (scale 0–10, higher scores indicate greater pain intensity). We selected three NRS types: average NRS (average pain intensity over the last 2 weeks), current NRS (current pain intensity), and most sever NRS (most severe pain intensity over the last 2 weeks).

#### Secondary outcome

Secondary outcomes consisted of mental and physical health-related quality of life by use of the 36-item short form health survey (SF36), which included physical and mental health summary (PCS and MCS) scales; we calculated the results based on Hong Kong-specific scoring algorithms [27]; anxiety was measured using the Generalized Anxiety Disorder-7 (GAD-7; range, 0–21; higher scores indicated greater severity) [28]; depressive symptoms were assessed using the Self-Rating Depression Scale (SDS; range, 25–100; higher scores indicated greater severity/minor depression:53–62; moderate depression: 63–72; major depression: over 72) [29]. Treatment adherence was indicated by the completion of the weekend exercise log: non-adherence was not completed once; incomplete adherence was completed less than 6 times, and complete adherence was completed all 6 times.

### Statistical analysis

We used intent-to-treat approach to analyze all available data at baseline, week 6, and week 18. We compared groups on baseline demographic and clinical characteristics using *χ*^2^ tests (or Fisher’s exact probability method) for categorical variables. For the continuous variable, normality tests were first tested. When the results fit, we used the independent *t*-test; when the results did not fit, we chose the rank sum test. Analyses of the primary and secondary continuous outcome variables were analyzed using two way repeated-measures ANOVA. When the results did not conform to the Mauchly test, we used Greenhouse-Geisser to proofread. The interaction effect was first tested. When the results were significant, between-group differences were tested at *α* = 0∙05 at each time point. When the results were not significant, the main effect of group and time was next tested. Otherwise, Bonferroni correction was applied at each time point, with *p*-values adjusted by multiplying the nominal *p*-value by the number of tests; partial eta-squared (*ƞ*^2^_p_) was reported to demonstrate the effect size. The outcome variable’s missing final values were replaced by the last known value before the participant lost to follow-up. All data were analyzed using IBM SPSS Statistics V.22.

## Results

**Fig. 2:**
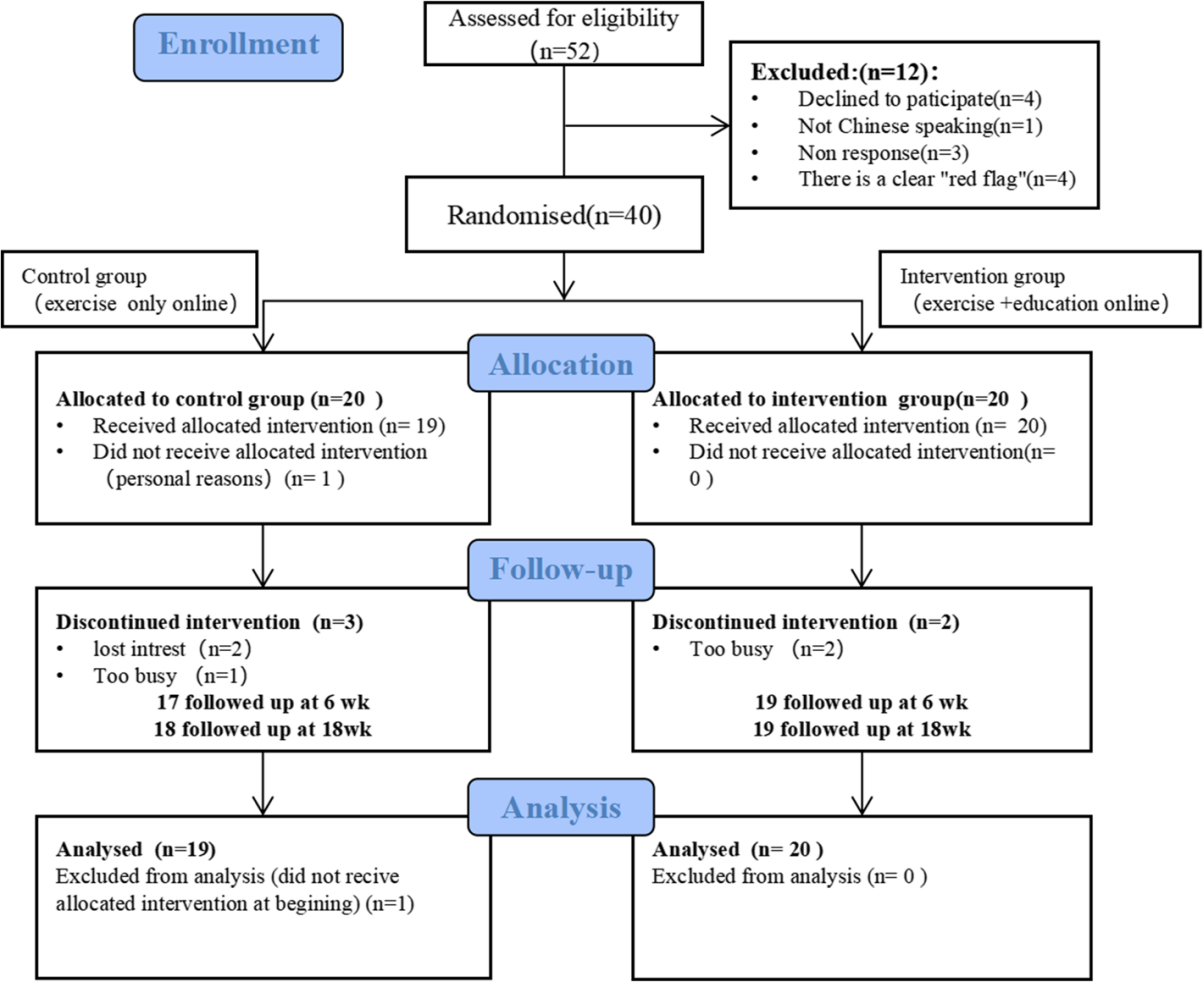
CONSORT diagram of study participation

A total of 52 participants were screened for eligibility, and 40 met eligibility criteria; they were randomized into two groups between November 2, 2020, and December 14, 2020, with the final follow-up on March 6, 2021 (Fig. 2). We found that most participants 28 (70%) chose massage for treatment only, 11 (27.5%) chose exercise for treatment, and 22 (55%) did not live in Guangzhou, including 1 (2.5%) who was studying abroad (Japan). The participants’ baseline characteristics are presented in Table 1. Participants’ educational attainment was generally high. RMDQ, current NRS, GAD-7, and MCS scores did not fit a normal distribution, so we used the rank sum test to compare. The intervention group’s degree of depression and physical health summaries were worse than the control group (Table 1), and the results showed significant differences. No significant differences were found in other baseline data. No adverse events related to the intervention or control group were noted.

**Fig. 3:**
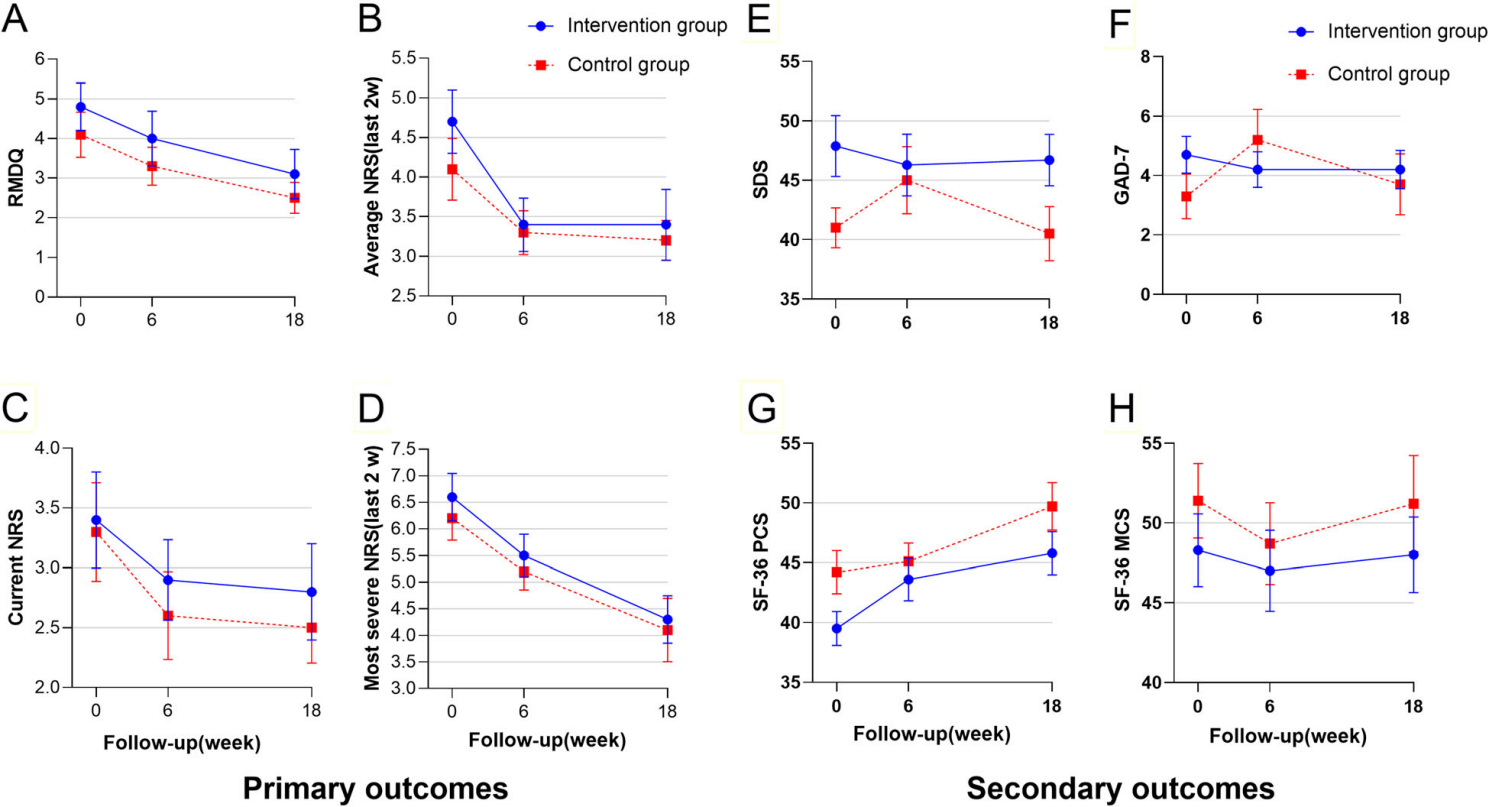
Changes in primary and secondary outcomes. The within group change (mean, SEM) of primary outcomes are shown for RMDQ (**A**), average NRS in the last 2 weeks (**B**), current NRS (**C**), and most severe NRS in the last 2 weeks. The secondary outcomes are shown for depression (**E**), anxiety (**F**), physical health summary scales (**G**), and mental health summary scales (**H**)

The primary outcome measures included function (RMDQ score) and pain (NRS score; Table 2, Fig. 3). Although the RMDQ and the NRS did not show significant interaction effects, a significant effect of time was found in both scores; the main effect of group did not show a significant difference. There were three levels of time factor, so post hoc analysis was made. Comparing the baseline, the effect size of the RMDQ score, the average NRS, and the most severe NRS was *ƞ*^2^ = 0.406, 0.262, and 0.423, respectively. Differences on the current NRS pain measure between groups did not reach statistical significance (*P* = 0.061).

**Table 2:** Primary and secondary outcomes

Measure by assessment Mean (SD)	Follow-up time			Main effect of time		Main effect of group		Interaction effects	
	Baseline	6-week follow-up	18-week follow-up	*F*_time_	*P*_time_	*F*_group_	*P*_group_	*F*_**Group × Time**_	*P*_**Group × Time**_
RMDQ									
Intervention group	4.8 (2.7)	4.0 (3.1)	3.1 (2.8)	**14.256**	**0.000**	0.813	0.313	0.043	0.952
Control group	4.1 (2.5)	3.3 (2.1)	2.5 (1.7)						
Average (NRS)									
Intervention group	4.7 (1.8)	3.4 (1.5)	3.4 (2.0)	**6.394**	**0.004**	0.515	0.478	0.477	0.624
Control group	4.1 (1.7)	3.3 (1.2)	3.2 (1.1)						
Current (NRS)									
Intervention group	3.4 (1.8)	2.9 (1.5)	2.8 (1.8)	3.034	0.061	0.187	0.668	0.065	0.937
Control group	3.3 (1.8)	2.6 (1.6)	2.5 (1.3)						
Most severe (NRS)									
Intervention group	6.6 (2.0)	5.5 (1.8)	4.3 (2.0)	**27.163**	**0.000**	0.457	0.503	0.089	0.891
Control group	6.2 (1.8)	5.2 (1.5)	4.1 (2.6)						
SDS									
Intervention group	47.9 (11.5)	46.3 (11.7)	46.7 (9.7)	1.179	0.313	2.580	0.117	2.670	0.078
Control group	41.0 (7.3)	45.0 (12.4)	40.5 (9.9)						
GAD-7									
Intervention group	4.7 (2.8)	4.2 (2.7)	4.2 (2.9)	1.529	0.224	0.104	0.749	3.106	0.052
Control group	3.3 (3.3)	5.2 (4.5)	3.7 (4.5)						
PCS (SF-36)									
Intervention group	39.5 (6.4)	43.6 (8.0)	45.8 (8.1)	**15.459**	**0.000**	2.456	0.126	1.283	0.282
Control group	44.2 (7.9)	45.1 (6.7)	49.7 (8.7)						
MCS (SF-36)									
Intervention group	48.3 (10.2)	47.0 (11.4)	48.0 (10.6)	0.860	0.420	0.790	0.380	0.115	0.877
Control group	51.4 (10.2)	48.7 (11.2)	51.2 (13.2)

The secondary outcome measures are also presented in Table 2 and Fig. 3. Only the PCS of SF-36 showed a significant effect of time. Comparing the baseline, the PCS scores were at 6 weeks (mean deviation, 2.454; 95% CI, − 0.201 to 5.109) but did not reach statistical significance (*P* = 0.078) and at 18 weeks (mean deviation, 5.823; 95% CI, 2.900 to 8.745). At the treatment end, anxiety and depression levels in the intervention group improved compared to the control group but not by week 18 (Fig. 3).

The treatment adherence rate is presented in Table 3. We found that more participants from the intervention group were willing to complete weekly exercise logs, and the difference was statistically significant.

**Table 3:** The treatment adherence

Group	NA number	IA number	CA number	The adherence rate *n* (%)
Intervention group (*n* = 20)	1	9	10	19 (95)
Control group (*n* = 20)	7	7	6	13 (65)
*P* (Fisher)				**0.044**

*NA* non-adherence, *IA* incomplete adherence, *CA* complete adherence

## Discussion

We found that both intervention and control groups significantly improved in short-term function at week 18, but m-health-based exercise (via plus education) did not improve participant outcome. The two groups significantly improved average and most severe pain in the last 2 weeks and the physiological functional aspects of quality of life during the course of the 18-week follow-up. As for the secondary outcomes, participants were able to use patient education to treat their anxiety and depression after treatment, but the relief did not last to week 18. Finally, the intervention group’s treatment adherence was significantly higher than that of the control group, and the results were statistically significant.

From the participants’ registration information, exercise had proved to be more effective than massage [30]; unfortunately, only a small percentage of our participants had tried exercise therapy. This outcome was similar to previous study results [31]. Patients with CLBP in under-resourced areas struggled to receive professional guidance, and our m-health-based study addressed this problem. In our study, most patients did not receive on-site treatment due to distance and time conflicts, but these inhibitions did not affect treatment progress. Additionally, through detailed participant communication, we found that the participants lacked understanding of low-back pain and maintained negative habits, such as sedentariness, so in the intervention group, we strengthened education and communication, with the aim of teaching the participants positive-intervention habits and self-management [32]. The results of the study by Chhabra et al. showed health applications are promising tools for improving pain and mood in patients with CLBP [33].  However, the mood of the participants in our study did not improve significantly. This is the point to be improved in our future research. Patient education improved treatment adherence in our study, which should be related to strengthening patients’ understanding of low back pain. CLBP patients seem to adapt better with education strategies in comparison to just guidance. However, the intervention group’s treatment effect did not show better improvement as compared to the control group, which may be due to the following reasons: this was a preliminary study with an insufficient sample size, and the patients’ severity and psychological levels in the intervention group were more serious than that of the control group. Therefore, the sample size should be expanded for future study.

The study strengths were that the study was a clinical trial without clinical sites [34], and participants could receive treatments anywhere and anytime [35]. Compared to other studies on low-back pain education [36, 37], our study was more convenient, efficient, and labor-saving effective with the use of Ding Talk. In our trial, participants wanted to exercise in their spare time based on the video, and they could ask questions at any time. The online-only recruitment and online-questionnaire collection also simplified the process and saved time, costs, and increased convenience to researchers. The main research site was located in the participants’ home, rather than the research center, thus saving costs. Additionally, the online-treatment therapies prevented COVID-19 exposure due to maintaining social isolation [38]. There is still much room for progress for m-health with the rapid development of information technology, and m-health-based exercise could be an alternative effective treatment for the CLBP patients, especially during COVID-19.

However, this study has some limitations. First, the participants were required to complete the exercise program without the supervision of a physical therapist, so we could not guarantee if the process completion was at quality standard. Second, the participant number was insufficient, and there was bias between the intervention and control groups on psychological indicator(s); the participants in the intervention group had poorer mental health than the control group. Third, the recruitment process may have selection bias, since the recruitment was made through a social network. Fourth, this study does not include an additional blank control group in order to provide as much benefit as possible to participants. Last, treatment adherence records may not be very accurate, due to technical deficiencies.

In future research, we should not only increase the patients’ psychological intervention to improve their mental health but also make full use of advanced information technology to increase the research quality. And we will consider increasing the blank control group and obtaining consent to patients by increasing financial input. We should adopt more useful educational measures in future research, such as the Pain Neuroscience Education [39]. Combined with psychology methods, such as online cognitive behavioral therapy (CBT) [40] or online mindfulness [41] for treatment, the treatment effect may improve more.

## Conclusion

In conclusion, m-health-based exercise guidance may be a convenient and effective method to treat CLBP. However, additional health education did not help more. In addition, the lack of a blank control group may have affected the results. Therefore, in order to further improve the research effect and accuracy, we need to improve the intervention methods (Such as adding psychotherapy) and add a no treatment control group in future studies.

## Data Availability

All data generated or analyzed during this study are included in this published article [and its supplementary information files].

## Abbreviations

M-health: Mobile health
CLBP: Chronic low-back pain
RMDQ: Roland and Morris Disability Questionnaire
NRS: Numeric Rating Scale
CSE: Core stability exercise
GAD-7: 7-Item Generalized Anxiety Disorder scale
SDS: Self-Rating Depression Scale
SF-36: 36-Item short form health survey
PCS: Physical health summary
MCS: Mental health summary
